# Flexibility of the N-Terminal mVDAC1 Segment Controls the Channel’s Gating Behavior

**DOI:** 10.1371/journal.pone.0047938

**Published:** 2012-10-23

**Authors:** Barbara Mertins, Georgios Psakis, Wolfgang Grosse, Katrin Christiane Back, Anastasia Salisowski, Philipp Reiss, Ulrich Koert, Lars-Oliver Essen

**Affiliations:** Department of Chemistry, Philipps University Marburg, Marburg, Germany; Virginia Commonwealth University, United States of America

## Abstract

Since the solution of the molecular structures of members of the voltage dependent anion channels (VDACs), the N-terminal α-helix has been the main focus of attention, since its strategic location, in combination with its putative conformational flexibility, could define or control the channel’s gating characteristics. Through engineering of two double-cysteine mVDAC1 variants we achieved fixing of the N-terminal segment at the bottom and midpoint of the pore. Whilst cross-linking at the midpoint resulted in the channel remaining constitutively open, cross-linking at the base resulted in an “asymmetric” gating behavior, with closure only at one electric field´s orientation depending on the channel’s orientation in the lipid bilayer. Additionally, and while the native channel adopts several well-defined closed states (S1 and S2), the cross-linked variants showed upon closure a clear preference for the S2 state. With native-channel characteristics restored following reduction of the cysteines, it is evident that the conformational flexibility of the N-terminal segment plays indeed a major part in the control of the channel’s gating behavior.

## Introduction

Voltage dependent anion channels (VDACs) are the most abundant proteins in the mitochondrial membrane with about 10 000 copies per cell [1]. VDACs mediate the flow of energy carrying molecules like ATP, ADP, pyruvate or succinate [2]. With their N-terminus providing a surface for interactions with pro- and anti-apoptotic proteins, they also become key players in the control of apoptotic signaling [3], [4], [5]. In lipid bilayer recordings, VDACs show multiple conductance states: a high conductance, referred to as the open state, and several low conductance states referred to as closed or partly open [6]. The low conductance states are usually reported at a current of 40–60% of that of the high conductance. In the high conductance state the channel is weakly anion selective, but exhibits weak cation selectivity when partly or fully closed [7].

Despite their extensive characterization, first insights into the VDAC 3D-structure were only recently obtained. Independently solved X-ray structures revealed an atypical 19 stranded β-barrel with an α-helix spanning the middle of the pore [3], [6], [7]. Based on the current structural data, it is thought that alignment of the α-helix with the barrel wall yields the “open” state [8]. Despite the availability of structural information, the complexities of the VDAC conductance states and the molecular mechanism underlying their transitions are poorly understood. Current hypotheses favor control of the opening/closure of the channel through a complete or partial movement of the N-terminal helix with its flexible hinge region (amino acids 19–25) into [9] or out of the pore [10]. Although an inward α-helical movement could control the channel’s activity through blockage, it fails to account for the channel’s ion selectivity, which an outward-movement model addresses more adequately [10]. None of the proposed models, however, has received strong experimental support. Recent work questioned the previous hypotheses and claimed that the N-terminal helix is not responsible for the modulation of the channel’s gating at all [11].

Here, we report the engineering of two mVDAC1 channel variants, which under oxidative conditions deviated strongly from the typical native channel gating: one that has lost its symmetrical response to the applied potential (V3C-K119C) and one which was trapped in its conducting state (A14C-S193C). We confirm formation of the disulfide bonds between the aforementioned residues by SDS-PAGE and/or peptide mass fingerprint spectrometry as well as planar lipid bilayer recordings, thus attesting the current structural models. In addition, we demonstrate that the α-helix plays a major part in the voltage gating process by undergoing a combination of two structural changes: one allowing the far N-terminus to flip into the channel, and a second requiring a re-organization of the α-helical residues. Beside the observed S1 state we further identify the presence of two well-defined low-conductance sub-states (S2A and S2B), which reinforce the flexibility of VDACs, and use the above mechanism to address aspects of the channel’s ion selectivity.

## Materials and Methods

### 1. Cloning and Site-directed Mutagenesis

Plasmid vectors, harboring the *murine* VDAC1 (mVDAC1) gene, were kindly provided by Dr Jeff Abramson (University of California LA). The *mVDAC1* gene was amplified by PCR, using the 5′ TTA ATA CTC GAG TTA TGC TTG AAA TTC CAG TCC TAG GC 3′ and 5′ ATA AAT CAT ATGGCC GTG CCT CCC AC 3′ primers, introducing the *Xho*I and *Nde*I restriction sites respectively. PCR products were *Xho*I/*Nde*I (*Fermentas*) digested, PCR cleaned (*Qiagen*) and cloned into pre-digested with the same enzymes and dephosphorylated (alkaline phosphatase, *NEB*) pET20b plasmid vector (*Novagen*). Ligations were performed with T4-ratio, according to the manufacturer’s instructions. Transformants were selected on an LB-ampicillin (100 µg.ml^−1^) (*Applichem*) containing plates.

For the construction of the Δ1–21 deletion variant, the *Xho*I/*Nde*I-flanked mVDAC1 gene was PCR amplified by primers 5′-TTA ATA CTC GAG TTA TGC TTG AAA TTC CAG TCC TAG GC-3′ and 5′-ATA AAT CAT ATG TGC GGC TTT GGC TTA ATA AAA CTT GAT TTG AAA AC-3′, the latter introducing an *Nde*I site with a Y22C mutation. The PCR product was digested with *Xho*I/*Nde*I, PCR cleaned (*Qiagen*), and ligated in a 3∶1 ratio with *Xho*I/*Nde*I digested and dephosphorylated pET20b vector. Transformants were selected on LB-ampicillin (100 µg.ml^−1^) (*Applichem*) containing plates.

The cysteinless mVDAC1-C127A-C232A construct was created by the QuickChange (*Stratagene*) PCR-based mutagenesis method, as previously described [12], [13], with the exception that *DpnI* treatment was prolonged relative to the manufacturer’s instructions. Mutagenic primers 5′-G CAC ATC AAC CTC GGG GCT GAC GTG GAC TTT GAC-3′and 5′-GTC AAA GTC CAC GTC AGC CCC GAG GTT GAT GTG C-3′ were used for the introduction of the C127A mutation. Having confirmed the identity of the C127A construct by DNA sequencing (*Qiagen*), the latter was subsequently used in a second PCR round for the introduction of the final mutation (primers 5′-CC AAG TAT CAG GTG GAT CCT GAT GCC GCC TTT TCG GCC AAA G-3′ and 5′-C TTT GGC CGA AAA GGC GGC ATC AGG ATC CAC CAG ATA CTT GG -3′).

Further mutagenesis was performed on the cysteine-free mVDAC1 plasmid using the 5′-CTT GGC AAG TCC TGC AGG GAT GTC TTC-3′ and 5′-GAA CAC ATC CCT GCA GGA CTT GCC AAG-3′ primers in the first round (A14C mutation), and 5′-CA GAG TTT GGT GGC TGC ATT TAC CAG AAG G-3′ and 5′-C CTT CTG GTA AAT GCA GCC ACC AAA CTC GT-3′ primers in the second (S193C mutation). Similarly for the construction of the V3C/K119C variant primers 5′-GAA GGA GAT ATC CAT ATG GCC TGC CCT CCC ACA TAC G-3′/5′-C GTA TGT GGG AGG GCA GGC CAT ATG GAT ATC TCC TTC-3′ and 5′-GGG TAC TGC AGG GAG CAC ATC AAC CTC GGC GCC GAC GTG-3′/5′-CCC ATG ACG TCC CTC GTG TAG TTG GAG CCG CGG CTG CAC-3′ were used. Identity of all constructs was confirmed by DNA sequencing (*Qiagen*).

### 2. Inclusion Body Production of mVDAC1 Variants

*Escherichia coli* BL21(DE3)-omp9 cells [F^-^, *ompT hsdS_B_* (r_B_^−^ m_B_^−^) *gal dcm* (DE3) Δ*lamB ompF::*Tn5 Δ*ompA* Δ*ompC* Δ*ompN*::Ω] [14] were transformed with pET-20 b harboring the native or mutagenized mVDAC1 gene without its signal sequence. Transformants were grown in LB medium containing ampicillin (100 µg.ml^−1^) and 1.0% (w/v) glucose at 37°C. Induction was performed by addition of 1 mM isopropyl-β-D-thiogalactopyranoside (IPTG) (*Gerbu Biotech.*) at an optical density (λ = 595 nm) of ∼0.6. Cells were allowed to grow for further 4 hours before harvesting (6400 *g*, 15 min, 4°C). Cell-pellets were solubilized in 50 mM Tris/HCl (pH 8.0), 100 mM NaCl and were subsequently disrupted by emulsification (Emulsiflex C5, Avestin). The crude cell extract was centrifuged (25402 g, 30 min, 4°C), the supernatant was discarded and the protein-containing pellet was washed three times in 50 mM Tris/HCl (pH 8.0), 100 mM NaCl, 10 mM EDTA, 2.5% (v/v) Triton-X-100 (Triton-TEN buffer) for removal of residual *E. coli* lipids, and three times in 50 mM Tris/HCl (pH 8.0), 100 mM NaCl (TEN buffer). The protein pellet was finally resuspended in TEN buffer, fast-frozen in liquid nitrogen and stored at −80°C until further use.

### 3. Protein Refolding and Purification

Protein refolding was accomplished by rapid dilution in a two-step process. First, inclusion bodies (10 mg ml^−1^) were denatured in 25 mM NaH_2_PO_4_/Na_2_HPO_4_ (pH 7.0), 100 mM NaCl, 1 mM EDTA, 10 mM DTT, 6 M guanidinium chloride. The denatured protein mixture was added drop-wise to 25 mM NaH_2_PO_4_/Na_2_HPO_4_ (pH 7.0), 100 mM NaCl, 1 mM EDTA, 2.22% (v/v) lauryldimethylamine-oxide (LDAO), allowing for a ten-fold dilution of the guanidinium chloride concentration, and the mixture was gently stirred overnight at 8°C. Following a centrifugation step (6400 *g*, 10 min, 4°C), for removal of insoluble aggregates, the supernatant was subsequently added drop-wise to 25 mM NaH_2_PO_4_/Na_2_HPO_4_ (pH 7.0), 10 mM NaCl, 1 mM EDTA, 1 mM DTT, 0.1% (v/v) LDAO (a further ten-fold dilution of the guanidinium chloride concentration), and the mixture was gently stirred overnight at 8°C for a further day. Finally the mixture was applied onto a pre-equilibrated Fractogel EMD-SE Hicap cation-exchange column (5 mL) (*Merck*) which was attached to an ÄKTA prime purification system (Amersham Biosciences). Elution was performed using a multi-segment gradient of 10–1000 mM NaCl in 25 mM NaH_2_PO_4_/Na_2_HPO_4_ (pH 7.0), 1 mM EDTA, 1 mM DTT and 0.1% (v/v) LDAO. The mVDAC1-containing fractions were pooled and concentrated using a 10-kDa Amicon concentrator (*Millipore*) and applied onto a Superdex 200 (*GE-Healthcare*) size-exclusion column (SEC) pre-equilibrated with 10 mM Tris/HCl (pH 8.0), 100 mM NaCl and 0.05% LDAO. SEC regularly yielded >95% pure protein as verified by SDS-PAGE. Size excluded mVDAC1 fractions were pooled and further concentrated. Protein concentrations were determined from the UV-Vis absorbance of tested samples at 280 nm (Nanodrop ND-1000, *peqLab*), using extinction coefficients as predicted by ProtParam [15]. Purified samples were stored at 4°C until further use.

### 4. Oxidation and Reduction of mVDAC1 Variants

For effective induction of disulfide cross-linking in mVDAC1 variants, purified proteins were treated with 100 mM copper-phenanthroline (CuX_2_Ph) as previously described [11], [16]. CuX_2_Ph reactions were quenched by addition of 0.5 mM EDTA. Disulfide bond reduction was mediated by addition of 10 mM DTT. To prevent potential interference of CuX_2_Ph or DTT with the subsequently performed assays, treated protein samples were further purified using an Amicon Ultra-0.5 mL Filter Device (*Millipore*) according to the manufacturer's instructions. The presence or absence of disulfide bonds was confirmed independently by non-reducing SDS-PAGE and peptide mass fingerprint spectrometry.

### 5. SDS-PAGE

Protein samples were resolved on 12% gels, using the Laemmli buffer compositions [17]. Homogeneity and purity were densitometrically determined. Gel-shift assays using CuX_2_Ph- and DTT-pre-treated samples indicated the presence and disruption of the disulfide cross-link, respectively.

### 6. Peptide Mass Fingerprinting

To cap free cysteines, 2-iodoacetamide (*Applichem*) was added in a final concentration of 1 mM to the mVDAC1 samples and stirred over night at 8°C. To remove free 2-iodo-acetamide the protein was precipitated using a four-fold excess of acetone and the samples were incubated for 2 hours at −20°C. Precipitated mVDAC1 was centrifuged (25402 g, 3 min, 4°C) and the pellets were washed with iced water and ice-cold ethanol. Pellets (341 µg each) were dried at 37°C, dissolved in 100 µL 10% acetonitrile and digested over night at 37°C with 10 µg sequencing grade trypsin (*Promega*) containing 25 µL 50 mM NH_4_HCO_3_ buffer at pH 8.0. For chromatographic separation of the tryptic peptides an 1100 Agilent HPLC system was used with a 150x3 mm Kinetex 2.6 µm C18 column (*Phenomenex*) at a temperature of 40°C and a flow rate of 0.2 mL/min. UV-detection of the peptides was carried out at 215 nm. The following gradient was applied by mixing water/0.1% TFA (solvent A) and acetonitrile/0.1% TFA (solvent B): 5% B for 10 minutes, linear increase to 60% B within 150 minutes, linear increase to 90% B in additional 5 minutes and then holding B at 90% for 10 minutes. Mass spectrometric detection of the peptides was done by online electrospray-ionisation-mass spectrometry (ESI-MS) with an LTQ-FT mass spectrometer (*ThermoScientific*). An FTMS scan was carried out in the mass range of 400–2000 m/z, followed by three automated ion trap MS/MS scans. The disulfide-bridged peptides were identified from comparisons of predicted and actual masses.

### 7. Single Channel Conductance Recordings

Single channel conductance recordings were performed using the Black Lipid Membrane (BLM) technique [18], [19], [20], [21], [22], [23]. A sample of *n*-decane-solubilized diphytanoyl phosphatidylcholine membrane mixture (25 mg/mL) was painted over a 200 µm circular hole, separating the chamber compartments (polystyrene cuvette: CP2A, bilayer chamber: BCH-22A, *Warner Instruments*) filled equally with BLM buffer [10 mM Tris/HCl (pH 7.4), 1 M KCl, 5 mM CaCl_2_]. Protein (7.5–15 µg) was added to one compartment beside the planar lipid layer and a voltage-gradient was imposed across the membrane whilst waiting for insertion of single channels. Generally, ±40 mV voltages were applied, electric current was recorded using a Multipatch 700B patch-clamp amplifier connected to a Digidata 1440A A/D converter and traces were visualized through the pClamp 10.2 software (*Axon Instruments*). To avoid noise convolution of the recordings and to minimize the possibility of channels reconstituting in an antiparallel manner (see results and discussion), we analyzed only the recordings corresponding to the spontaneous insertion of up to five channels. Data were collected at 5 kHz and sampled at 200 Hz for further analysis. For each measurement, high and low conductance states were defined relative to a clear baseline, and their corresponding differences to the baseline were evaluated by the software. Linear regressions were performed by Origin 7 (*Origin Lab*). For every determined conductance value, 15–84 separate measurements were averaged at different voltage steps. Statistical tests were performed using the GraphPad (*GraphPad Software*) online calculators. Note that values are given as mean ± SEM, unless otherwise stated.

## Results

### 1. CuX_2_Ph Induced Cross-linking in mVDAC1 Variants Validates the 3EMN 3D-Structure

**Figure 1:**
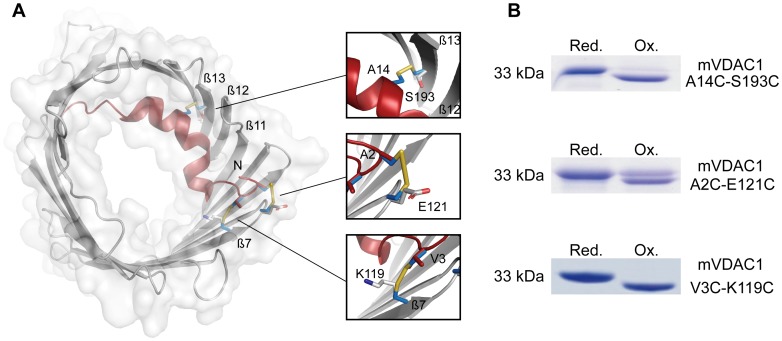
Cysteine mutagenesis on mVDAC1 and cross-linking of the N-terminal segment to the barrel wall. A) Cartoon representation of mVDAC1, based on the 3EMN structure [3], viewed perpendicular to the membrane plane. Location of the engineered cysteines and expected formation of disulfide bonds are shown as inlays. B) 12% Coomassie stained, non-reducing SDS-PAGE analysis of native and engineered mVDAC1 variants. Proteins were pre-treated with to copper-phenanthroline (oxidized) or DTT (reduced) as described in Materials and Methods. Increased electrophoretic mobility of protein bands indicated formation of disulfide cross-links. Following reduction by 10 mM DTT, “linearized” proteins migrated slower than their cross-linked counterparts.

Basing our construct design (Figure 1A) on the murine VDAC1 structure (3EMN, [3]) we found that covalent fixing of the N-terminal segment by engineering disulfide bindings to the porin’s inner wall was favorable. Following the successful refolding and purification of mVDAC1 variants containing appropriate cysteine mutations at the positions A2-E121, A14-S193 and V3-K119, disulfide-bridge formation was confirmed by denaturing SDS-PAGE electrophoresis. Reduced double cysteine variants migrated generally slower and closer to their actual molecular mass than their non-linearized, cross-linked counterparts (Figure 1B). Of the investigated variants, only A2C-E121C partly resisted the CuX_2_Ph treatment (Figure 1B), as it failed to oxidize completely, implying that formation of the corresponding bridge here was structurally disfavored. In addition, peptide mass fingerprinting of the oxidized A14C-S193C variant confirmed quantitative disulfide formation, by detection of the S13-R15/T175-Y197 fragment (Figure S1). The corresponding fragment of the V3C-K119C variant could not be detected, most likely due to lack of ionization. As confirmed by SDS-PAGE, the fully reduced mVDAC1 variants had a limited half-life of ∼22 hours (Figure S2) in the presence of air and hence showed a strong tendency towards oxidation. In essence, and in agreement with previously published works [11], our observations increased confidence that the 3EMN model [3] corresponds to that of the native and biologically most relevant open channel conformation.

### 2. Closure of the Native mVDAC1 Channel Occurs through at Least Two Low-conductance States

**Figure 2:**
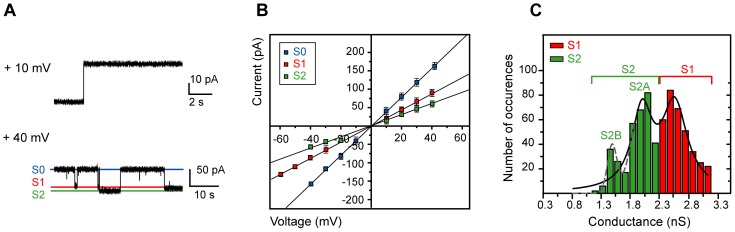
The gating profile of mVDAC1 shows switching events between an open (S0) and two closed (S1 and S2) states. A) Representative traces of the mVDAC1 gating activity at +10 and +40 mV. The observed S0, S1 and S2 states, in the +40 mV trace, are indicated by blue, red and green lines respectively. B) Ohm-plot used for the determination of the conductance values of the corresponding states. Reconstituted native mVDAC1 responded linearly to application of both positive and negative potentials, alternating between an open (S0; blue) and two major (S1; red and S2; green) closed states. Determined conductances were: S0; 3.94±0.04 nS (*n* = 680), S1; 2.61±0.01 nS (*n* = 386) and S2; 1.90±0.06 nS (*n* = 294). Bars represent standard errors of a minimum of 15 replicates per measurement. C) Histogram of occurrences of low conductance values for native mVDAC1. The sum of two Gaussians indicated clearly the presence of two low conductance states for the native channel: S1 (red) and S2 (green). S1 and S2 conductances were different not only with respect to each other (*P* = 10^−4^), but also with respect to the mean closed state conductance derived from the single Gaussian fit (Table S1). Applying the sum of two Gaussians to the S2 state (R^2^∶0.98) resulted in the identification of an additional population (S2B; dashed grey line), suggesting that S2 is the composite of two low conducting S2A (2.03±0.01 nS, *n* = 224) and S2B (1.48±0.02 nS, *n* = 70) sub-states (Table S1). Values are given as mean ± SEM.

Native mVDAC1 exhibited the well observed [3], [4], [7], [24], [25], [26], [27] and expected gating behavior, responding linearly to the application of both positive and negative potentials, alternating between high and low conductance states (Figure 2A). In the open state (S0), the porin exhibited a conductance similar to those previously described (Table 1). When we calculated the mean of the low conductance state data (green and red squares, Figure 2B) we obtained a value of 2.34±0.06 nS (*n* = 680) (Table S1). The latter conductance was ∼1.4-fold different to that reported by Ujwal *et. al.* [3] (Table 1). Thinking that this difference could not solely stem from random error, we attempted fitting the distribution of the low conductance data to the sum of two Gaussians (Figure 2C, Table S1). The sum of the two distributions worked better than the single Gaussian (Table S1) and enabled a clear identification of two closed states: a S1 (exhibiting 66% of the conductance of the S0 state) and a S2 (exhibiting 48% of the conductance of the S0 state) (Figures 2B, 2C). Interestingly, the determined S2 conductance value (1.90±0.06 nS; *n* = 294); Figures 2B, 2C) is in agreement with the values previously reported for channel closure (Table 1). Student t-tests were performed to statistically validate the differences between the observed conductances for 1) the S1 (*n* = 386) and S2 (*n* = 294) closed states and 2) for each closed state and the mean closed state (*n* = 680) derived from the single Gaussian fit (Table S1). S1 and S2 conductances were different not only with respect to each other (*P* = 10^−4^), but also with respect to the mean closed state conductance derived from the single Gaussian fit (Table S1). Furthermore, the S2 population (0.80–2.20 nS) indicated a double Gaussian distribution, revealing the presence of an additional low-conductance sub-state, S2B (Figure 2C). Hence, the conductance of the closed conformation is a composite of at least three separate, low conducting states, S1, S2A and S2B (Table S1). The average dwell time of the channel in one state before switching to the next was 10.34±1.65 s (*n* = 310) (Figure S3).

**Table 1:** Reported conductance values for VDACs following their reconstitution in planar lipids.

Protein	Reference	OpenState (nS)	Closed state (nS)	Salt composition
mVDAC1	Ujwal *et al.* [3]	3.70±0.40^a^	1.70±0.20^a^	1 M KCl5 mM CaCl_2_
mVDAC1	Abu-Hamad *et al*. [4]	4.00	ND	1 M KCl5 mM CaCl_2_
Δ1-26-mVDAC1	Abu-Hamad *et al*. [4]	4.00	–	1 M KCl5 mM CaCl_2_
hVDAC1	Shanmugavadivu *et al*. [26]	4.50±0.50^a^	ND	1 M NaCl
hVDAC1	Hiller *et al*. [7]	3.90±1.50^a^	ND	1 M KCl
hVDAC1	Engelhardt *et al*. [24]	∼ 5.00	2.00	1 M KCl
NcVDAC1	Popp *et al*. [25]	4.0	2.00	1 M KCl
rVDAC	Colombini *et al.* [27]	4.50	N D	1 M KCl
ScVDAC	Colombini *et al*. [27]	4.50	ND	1 M KCl
mVDAC1		3.94±0.04^b^(S0)	2.61±0.01^b^(S1)1.90±0.06^b^ (S2)	
Δ1-21mVDAC1-Y22C		4.00±0.04^b^(S0)	–	
mVDAC1-C127A-C232A		3.78±0.09^b^(S0)	2.55±0.12^b^ (S1)1.78±0.09^b^(S2)	
mVDAC1-A14C- C127A- S193C -C232A (oxidized)	This work	3.74±0.09^b^(S0)	1.85±0.07^b^low probability(S2)	1 M KCl5 mM CaCl_2_
mVDAC1-A14C- C127A- S193C -C232A (reduced)		3.75±0.12^b^(S0)	2.55±0.12^b^ (S1)1.67±0.09^b^(S2)	
mVDAC1-V3C-K119C- C127A-C232A (oxidized)		3.76±0.10^b^(S0)	1.77±0.09^b^(S2)	
mVDAC1-V3C-K119C- C127A-C232A (reduced)		3.98±0.04^b^(S0)	∼2.70 (S1)2.05±0.13^b^(S2)	

a values given with their standard deviations; ^b^ values given with their standard error;. ND, not determined.

### 3. The Δ1-21-mVDAC1-Y22C (Δ21-mVDAC1) Deletion Construct Shows a Clear Preference for the Open State

**Figure 3:**
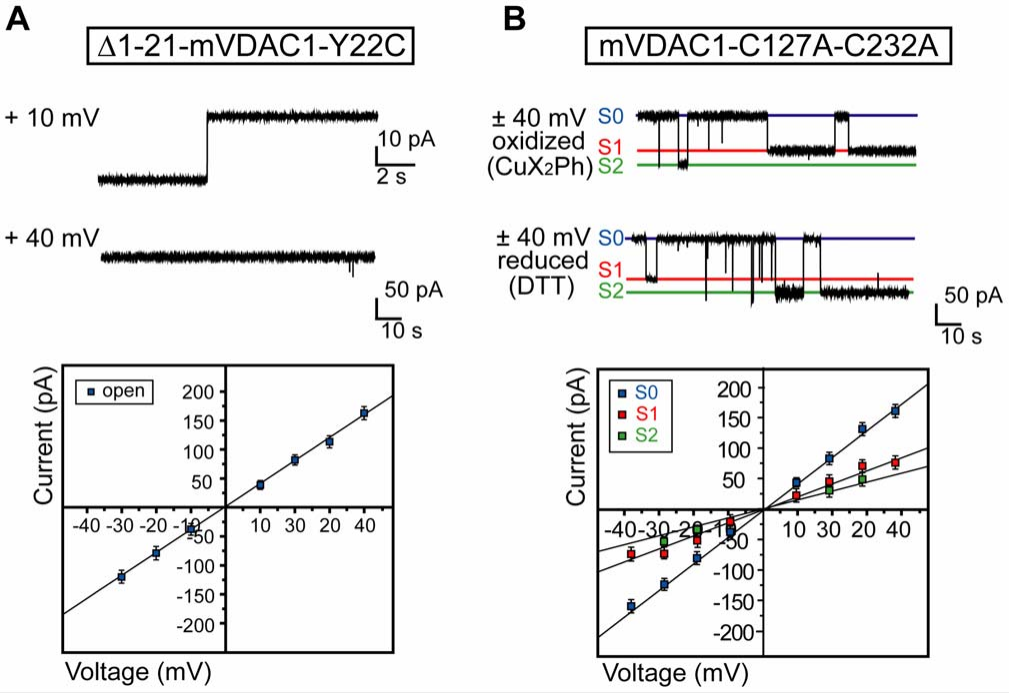
Gating profiles of reconstituted Δ1-21-mVDAC1-Y22C and cysteinless mVDAC1 variants . A) Top panel: representative traces of the Δ1-21-mVDAC1-Y22C gating activity at +10 and +40 mV. At +10 mV, a single channel reconstituted into the artificial membrane and conducted in the open state. At +40 mV the channel remained constitutively open. Bottom panel: Ohm-plot used for the determination of the conductance of the Δ1-21-mVDAC1-Y22C channel. The reconstituted channel responded linearly to application of both positive and negative potentials and exhibited a conductance of 4.00±0.04 nS (*n* = 20). This conductance was similar to that of the S0 state of the native protein (*P* = 0.80). B) Top panel: representative traces of the cysteinless variant at ±40 mV, following CuX_2_Ph or DTT pre-treatment (materials and methods). The gating behavior of the cysteinless variant was redox independent. Hence, the channel exhibited open (S0; blue) to closed (S1; red, S2; green) state transitions similar to those of the native channel. Bottom panel: Ohm-plot used for the determination of the conductance values of the corresponding states. Determined conductances were: S0; 3.78±0.09 nS (*n* = 28), S1; 2.55±0.12 nS (*n* = 24) and S2; 1.78±0.09 nS (*n* = 12). These conductances were similar to those of the native protein (S0; *P* = 0.42, S1; *P* = 0.24 and S2; *P* = 0.69). Bars represent standard errors of a minimum of 3 replicates per measurement. Values are given as mean ± SEM.

To prove the dominant role of the N-terminal α-helix in any gating behavior of mVDAC1, we analyzed the N-terminal truncation variant Δ21-mVDAC1. Current recordings were obtained following the reconstitution of the Δ21-mVDAC1 construct in planar lipid membranes (Figure 3A). Upon application of +10 mV, single channels spontaneously reconstituted in the artificial bilayer in the open state and remained constitutively open (Figure 3A), exhibiting a conductance of 4.00±0.04 nS (*n* = 20) in agreement with the reported observations of Abu-Hamad *et al*. [4] and De Pinto *et al*. [28]. T-test comparison of the S0 state conductances for native and Δ21-mVDAC1 proteins suggested that the compared values were similar (*P* = 0.80; sample size for native protein *n* = 680).

### 4. The Native and Cysteinless Channels Share the Same Gating Characteristics

The cysteinless variant (C127A-C232A) displayed native-like channel behavior, occupying the open S0 state with a conductance of 3.78±0.09 nS (*n* = 28, *P* = 0.42), the S1 state with a conductance of 2.55±0.12 nS (*n* = 24, *P* = 0. 24) and the S2 closed state with a conductance of 1.78±0.09 nS (*n* = 12, *P* = 0.69) (Table 1, Figure 3B). Note that the statistics provided compare the observed conductance states of the cysteinless variant with the corresponding conductance states of the native protein. The sample sizes for the native protein were: S0 (*n* = 680), S1 (*n* = 386) and S2 (*n = *294). Representative traces of the cysteinless channel are provided (Figure 3B). As expected, the behavior of the cysteinless variant was redox independent, since neither CuX_2_Ph nor DTT treatment affected its gating characteristics (Figure 3B).

**Figure 4:**
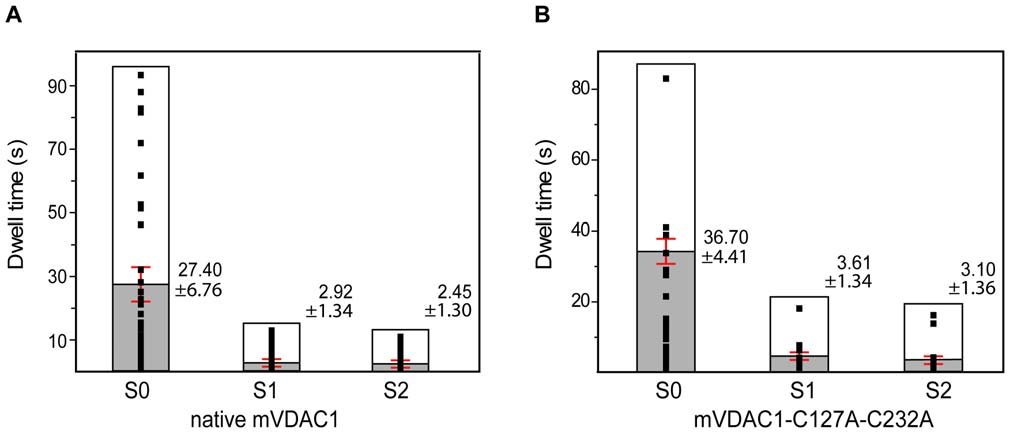
Dwell times for the individually attained conducting states by native and cysteinless mVDAC1 channels. S0, S1 and S2 conducting states were identified from collected traces, based on the values of Table 1 (±5%). A) Native mVDAC1 occupied the S0 state (27.40±6.76 s, *n* = 137) almost 10-fold longer (*P*≤0.0053) than the S1 (2.92±1.34 s, *n* = 92) and S2 (2.45±1.30 s, *n* = 81) states. Occupancy of both S1 and S2 was similar (*P* = 0.80). B) Cysteinless mVDAC1 occupied the S0 state (36.70±4.41 s, *n* = 14) also ∼10-fold longer (*P*≤10^−4^) than the S1 (3.61±1.34 s, *n* = 10) and S2 (3.10±1.36 s, *n* = 13) states. Occupancy of both S1 and S2 was similar (*P* = 0.80). State-to-state dwell time comparisons between native and cysteinless channels showed no differences (S0; *P* = 0.66, S1; *P* = 0.87 and S2; *P* = 0.84). Values are given as mean ± SEM.

The average dwell times of both native (10.34±1.65 s, *n* = 310) and cysteinless (10.45±5.06 s, *n* = 37) constructs were similar (*P* = 0.98) (Figure S3; Note that the average dwell times indicate the lingering in any state before transition to the next. Consequently, average dwell times correspond to switching events from and to all states). Furthermore both channels dwelled at the individual states for the same lengths of time (S0; *P* = 0.66, S1; *P* = 0.87 and S2; *P* = 0.84, Figure 4). Accordingly, any putative effects in the gating behavior of further modified mVDAC1 variants can only be attributed to the incorporation of the corresponding mutations and the disulfide bond-mediated immobilization of the N-terminal segment.

### 5. Disulfide Bond Formation in the A14C-S193C-mVDAC1 Double Mutant Favors Occupancy of the S0 and S2 States

**Figure 5:**
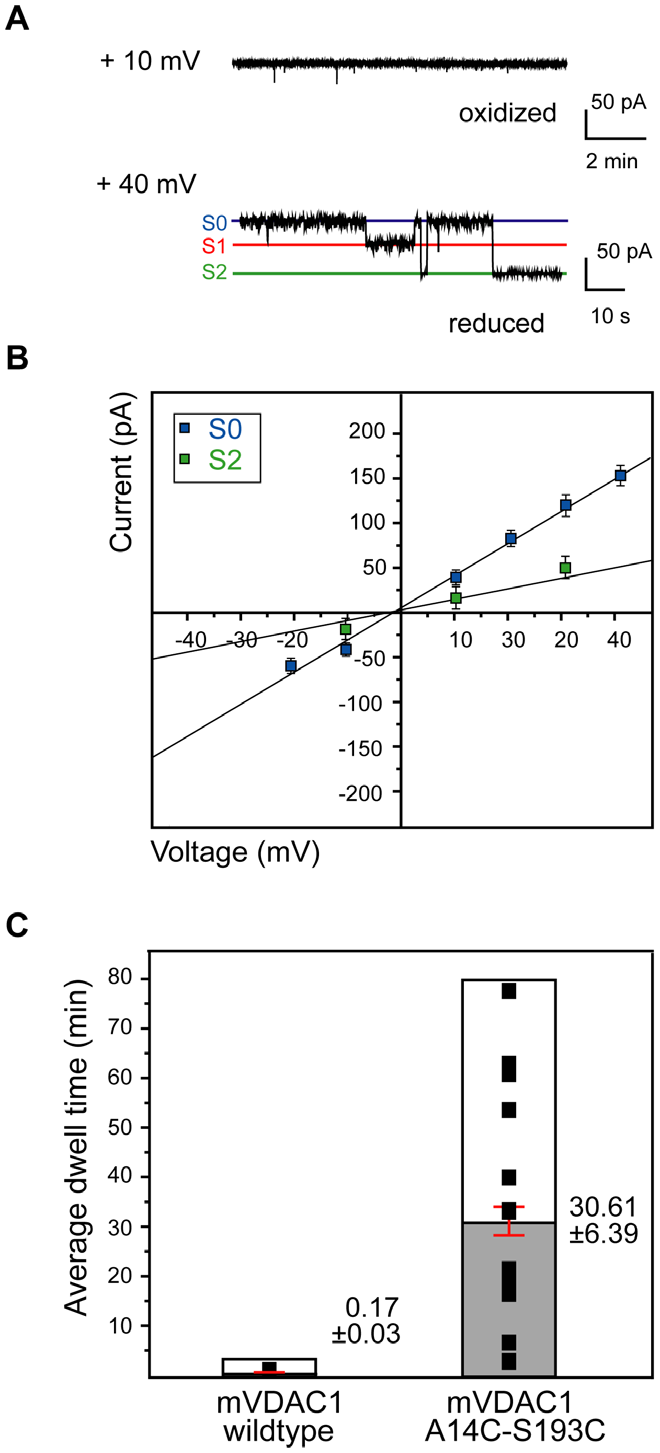
Cross-linking at the mid-point of the pore results in a channel “trapped” in its open S0 state. A) Representative traces of the A14C-S193C-mVDAC1 gating activity at +40 mV, following CuX_2_Ph or DTT pre-treatment (materials and methods). Pre-treated with DTT and subsequently reconstituted A14C-S193C-mVDAC1 exhibited the expected native-like channel gating. The oxidized mutant however, appeared to remain constitutively open (92% of the collected traces) with relaxation in the closed S2 state being strongly disfavored (8% of the collected traces). B) Ohm-plot used for the determination of the conductances of the observed states in the gating transitions of the A14C-S193C-mVDAC1 channel. The open state (blue squares) conductance of this variant was 3.74±0.09 nS (*n* = 30) and was similar to that of the native protein (*P* = 0.30). When the cross-linked channel relaxed in a closed state (green squares) the determined conductance was 1.85±0.07 nS (*n* = 14), which was similar to the S2 state conductance of the native protein (*P* = 0.86). Bars represent standard errors of a minimum of 3 replicates per measurement. C) Average dwell times of native and oxidized A14C-S193C mVDAC1. Mean times are shown in grey (native channel; *n* = 310 and A14C-S193C oxidized variant; *n* = 14) with red bars indicating the standard error. The mean dwell time (30.61±6.39 min) for the oxidized mutant channel, before switching to a different state, was prolonged 180-fold relative to that of the native channel (0.17±0.03 min). T-tests indicated that the observed difference was statistically significant (*P* = <10^−4^). Values are given as mean ± SEM.

As expected, fully reduced A14C-S193C-mVDAC1 displayed native-like channel characteristics alternating between a 3.75±0.12 nS high (S0) and a 1.70±0.1 nS low (S2) conductance states (Figure 5A, Table 1). The observed state conductances were similar to those of the native protein (S0; *P* = 0.32, S1; *P* = 0.22 and S2; *P* = 0.30. Sample sizes were *n* = 30 for every reduced-mutant state. Sample sizes for the native protein were as above). Disulfide cross-linking by CuX_2_Ph after reconstitution of this as well as other variants in planar lipid bilayers failed, resulting in native-like channel recordings. However, pre-oxidation and subsequent voltage-driven lipid reconstitution resulted in current recordings supporting almost a lock in the S0 conductive state (3.74±0.09 nS; *n* = 30) (Figures 5A, 5B). The attained S0 conductance of the oxidized mutant was similar to that of the native protein (*P* = 0.30). In the minority of collected traces (30/358), and always following extended lingering in the S0 state, the channel finally relaxed to a low conductance state (1.85±0.07 nS, *n* = 14), equivalent to S2 of native mVDAC1 (*P* = 0.86), omitting the S1 state and failing to reopen (Figure 5B). Furthermore, the average dwell time for the oxidized A14C-S193C variant in the S0 state (30.61±6.39 ≡ 1836.60±372.91 s, min, *n* = 14) was 180 times longer (*P*<10^−4^) in comparison to that of the native mVDAC1 (0.17±0.03 min ≡ 10.34±1.65 s, *n* = 310) (Figures 5C, S3).

### 6. Disulfide Bond Formation in the V3C-K119C-mVDAC1 Variant Enables Closure of the Channel Under One Type of Potential Depending on its Orientation in the Planar Lipid Bilayer

**Figure 6:**
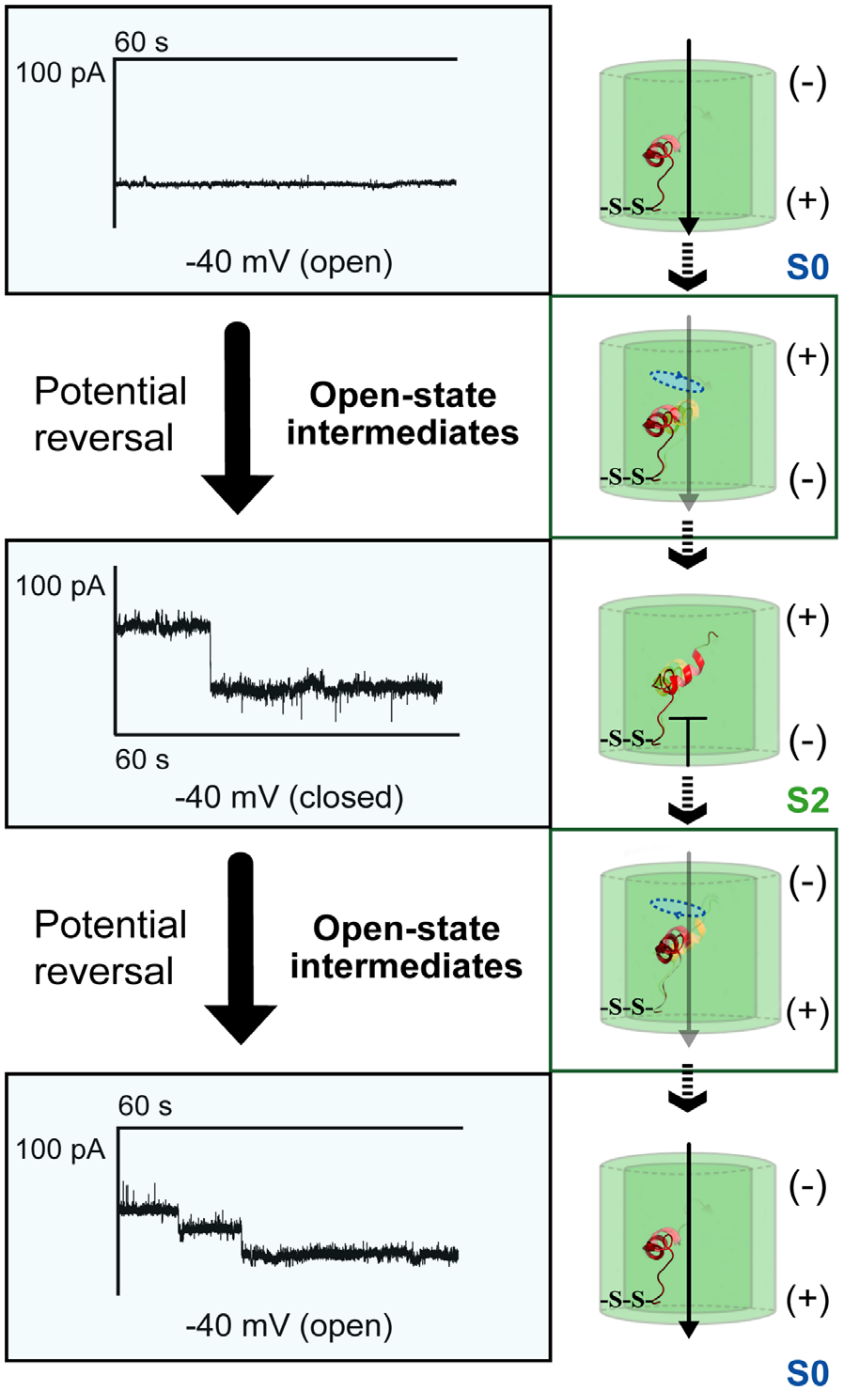
Cross-linking at the base of the pore abolishes symmetric voltage gating. CuX_2_Ph pre-treated and subsequently reconstituted V3C-K119C-mVDAC1 conducted in the open state upon application of both negative and positive potentials (e.g. −40 mV) but closed only at one set of potentials (e.g. +40 mV), depending on its orientation in the lipid layer. As indicated in the corresponding traces (one step closing, two-step opening) and the adjacent cartoons, the gating behavior was dynamic in both directions, and was probably mediated by a series of short-lived intermediates (Note that the conductances of these intermediates exhibited ≥5% difference with respect to the conductances of the S0 and S1 states). The probable movement of the 5–14 residue part is sketched (dashed blue ellipticity). Cross-linking at the base of the channel is designated –S-S-. Polarity of the membrane is indicated.

**Figure 7:**
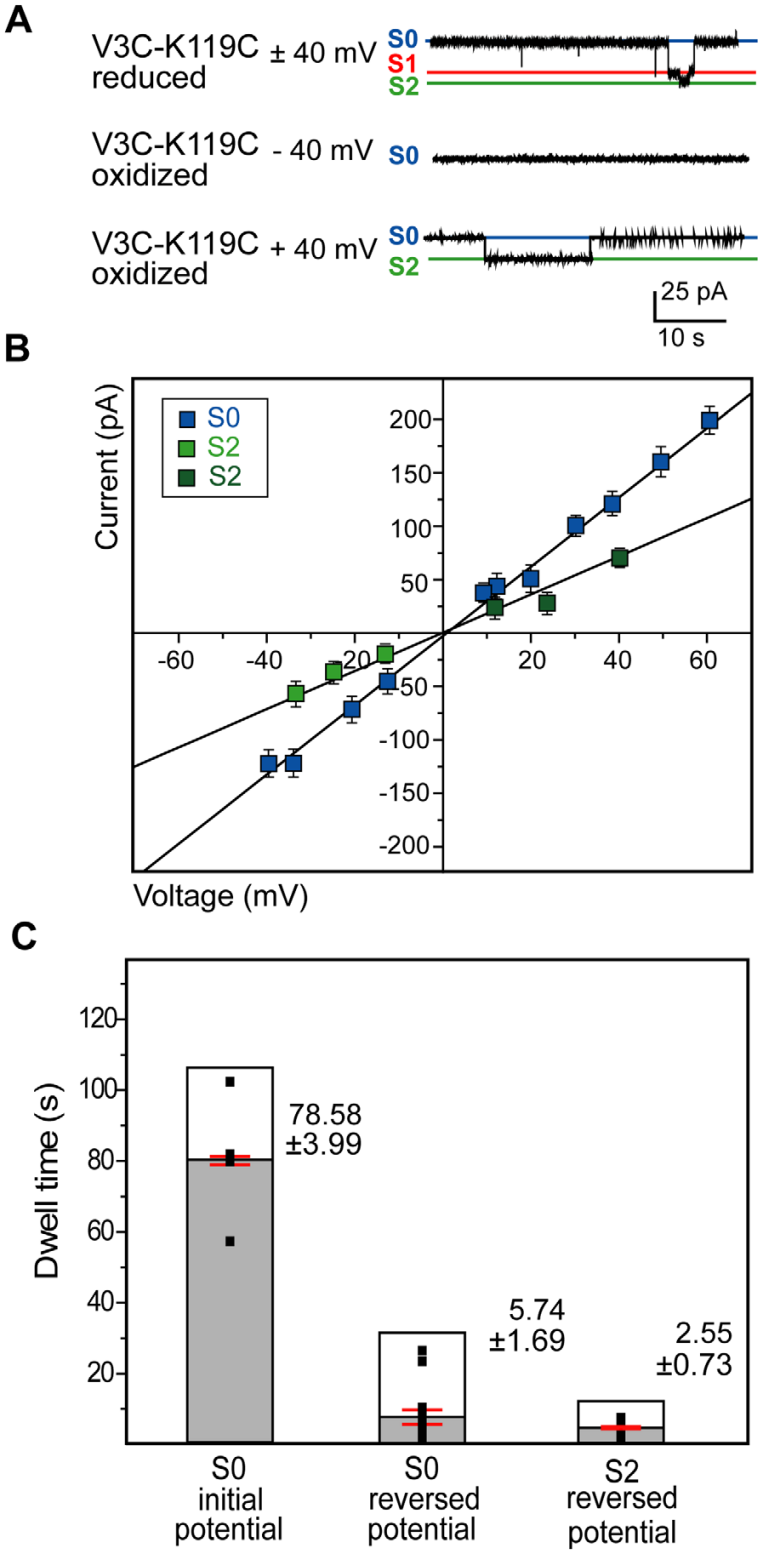
Gating profile and dwell times of reconstituted V3C-K119C-mVDAC1. A) Representative traces of the V3C-K119C-mVDAC1 gating activity, following CuX_2_Ph or DTT pre-treatment (materials and methods). Under reducing conditions, reconstituted V3C-K119C-mVDAC1 responds symmetrically to application of positive and negative potentials alternating, like the native channel, between an open (S0; blue) and at least two defined (S1; red and S2; green) closed states. When cross-linked and upon voltage application (−40 mV) the channel inserts itself in the planar lipid membrane and conducts in the open state. Application of the reverse potential (+40 mV) brings closure (one step) and the channel reopens only when −40 mV is applied. B) Ohm-plot depicting the conductance of the channel in the open state (blue) and closure of the channel only upon application of the reverse potential (light green and dark green represent closure upon reversal of positive and negative potentials respectively, based on the channel’s initial direction of insertion in the membrane). The open state (blue squares) conductance of this variant was 3.76±0.10 nS (*n* = 77) and was similar to that of the native protein (*P* = 0.15). Upon reversal of the potential, the determined closed state conductance (1.77±0.09 nS, *n* = 30) was similar to the S2 state conductance of the native protein (*P* = 0.49). Bars represent standard errors of a minimum of 3 replicates per measurement. C) Dwell times for oxidized V3C-K119C-mVDAC1. Mean times are shown in gray with red bars indicating the standard error. Values are given as mean ± SEM. The cross-linked V3C-K119C variant, for a chosen initial potential, assumes the S0 state with a dwell time of 78.58±3.99 s (*n* = 8). With potential reversal, the channel lingers in the S0 state for 5.74±1.69 s (*n* = 17) before switching to the S2 state, which it only assumes for 2.55±0.73 s (*n* = 7) as another transition to S0 follows (+40 mV trace in panel A).

Pre-oxidation and subsequent voltage-driven lipid reconstitution of the V3C-K119C mutant resulted in current recordings which showed channel opening under both positive and negative potentials (306 traces), but closure only under one or the other (300 traces) (Figures 6, 7A and 7B). Pre-reduction of the disulfide bond restored native gating characteristics (Figure 7A) with: S0 at 3.75±0.12 nS (*n = *30, *P* = 0.32), S1 at 2.55±0.12 nS (*n* = 20, *P* = 0.24) and S2 at 1.67±0.09 nS (*n* = 20, *P = *0.32), where *P* provides the strength of similarity to the corresponding conductance states of the native protein (native channel conductances - Table 1 - and sample sizes were as above).

The type of potential required for the cross-linked channel’s closure was determined by the orientation of the protein within the BLM set-up. Thus, channels incorporating into the bilayer upon application of a positive potential conducted in the open state, and showed decreased voltage-dependent conductance (closed state) upon voltage reversal (Figure 7B). Likewise, channels incorporating into the bilayer at negative potentials conducted in the open state until the opposite voltage was applied (Figure 7B). The first disulfide-bridged V3C-K119C channel assumed a random orientation upon its reconstitution into the lipidic environment of the BLM set-up. Random channel orientation, following the reconstitution of membrane proteins into artificial bilayers, has been reported for the outer membrane porin G from *E. coli* [29], yeast VDAC [30], bacterial sodium channels [31] and pore forming peptides [32]. Though the biophysical details of these random insertions have not been fully resolved, it appears that depending on the lipid composition [30] and when protein is applied to the *cis* compartment (high salt concentration), a slightly more positive voltage on the *cis* side enables primary adsorption of the protein to the bilayer. Reconstitution is then completed when a full *trans*-negative voltage is applied. Subsequently reconstituted mVDAC1 channels (up to five), exhibited auto-directed insertion, in harmony with earlier observations [30], [33], [34], [35], probably due to a reduction of the energy barrier to subsequent insertions. This autodirected insertion ensured that the oxidized channels would not reconstitute in an anti-parallel manner. Had that been the case, one of the two would have been open in one voltage sign and the other in the opposite, a situation which would create the illusion of symmetrical voltage-dependent conductance changes. The oxidized A14C-S193C variant exhibited an open state S0 conductance of 3.76±0.10 nS (*n* = 77), similar to that of the native channel (*P* = 0.15; S0 conductance and sample size for the native protein were as above) (Figure 7B). The most-attained low-energy closed state (1.77±0.09 nS, *n* = 30) was similar to the S2 state of the native channel (*P* = 0.49; S2 conductance and sample size for the native protein were as above) (Figure 7B).

Restraining the putative mobility of the first three amino acids, introduced upon potential reversal a “slow-motion” effect in the channel’s gating behavior, with open-to-closed state transitions extending the channel’s average dwell time to 78.58±3.99 s (*n* = 8) with respect to the native protein (10.34±1.65 s, *n* = 310) (*P*≤10^−4^) (Figure S3). In 95% of the recorded events, the S1 state was avoided and S0-to-S2 transitions involved additional intermediates, with conductances exhibiting ≥5% difference to S0 and S1 conductances. S0-to-S1 transitions occurred only in ≤5% of the events and were independent of S2. The dwell-times of the channel in each one of the observed states are provided (Figure 7C). The cross-linked V3C-K119C variant, for a chosen initial potential (−40 mV), assumes the S0 state with a dwell time of 78.58±3.99 s (*n* = 8) (Figure 7C). Note that for the transition to the next state the potential has to be reversed otherwise the cross-linked mutant channel would remain open. With the reversal (+40 mV), the channel lingers in the S0 state for 5.74±1.69 s (*n* = 17) before switching to the S2 state (Figures 7A, 7C). The channel assumes the S2 state for 2.55±0.73 s (*n* = 7), before another transition to S0 occurs (Figures 7A, 7C). To date, this is the first VDAC1 mutant to exhibit non-symmetrical voltage responses with both open-to-closed and closed-to-open transitions clearly occurring through a series of low-conducting sub states.

## Discussion

VDACs have been the subject of extensive studies and lively discussions concerning their structural organization and gating mechanism [1], [11]. The recently published mVDAC1 and hVDAC1 3D-structures fueled further debates [36], [37] because they revealed a) a number of β-strands atypical for porins b) the formation of two parallel β-strands between β19 and β1 [3], [6], [7] and c) an alignment of the N-terminal α-helix with the inner pore wall [3] or at least a localization in the pore’s interior [7]. VDACs are capable of alternating between high and low conductance states, representing open and closed channel conformations respectively [2], [3], [27], [38], [39], [40]. Based on past studies, it has been argued that crystal structures represented the open state of the pore while the NMR structure represented the closed state [8]. Brownian calculations and molecular dynamics simulations have revealed the dependence of the channel’s ion selectivity on salt concentration, with low salt concentrations favoring the permeability of anions in the open state [41], [42], [43], [44]. The mechanistic details underlying the channel’s gating behavior and ion selectivity however, still remain largely unclear.

In this work, the 3EMN crystal structure [3] was used as a template for the design and construction of mVDAC1 double cysteine comprising variants. Formation of disulfide bridges in all our tested variants (Figure 1, Figure S1) attested the proposed 3D-model. Colombini disputed the organization of mVDAC1 in detergent-micelles, and consequently in the crystal structure, proposing instead a refolding mechanism involving a 19- to 13-strand transition [36]. This transition, however, should disfavor topologically the state alterations of our cross linked mVDAC1 variants. Nevertheless these variants exhibit gating, characteristic of S0-to-S2 transitions, albeit with lowered probability. In harmony with previous BLM and patch-clamp measurements [3], [4], [7], [24], [25], [26], [27], [43], [45] these results provide further support to the arguments of Hiller and co-workers that the 19-stranded 3D-structure represents indeed the biologically relevant open state [37].

To address the involvement of the N-terminal segment in the channel’s gating behavior we characterized the Δ21-mVDAC1 deletion variant. In agreement with previous observations [25], [28], our mVDAC1 deletion variant produced current recordings characterized by some flickering. Popp *et al*. [25], reported a preference of the Δ3–20-mVDAC1 for a closed state and, since Δ2–12 exhibited native-channel characteristics, proposed a role for the N-terminal fragment in stabilization of the open state. Our data, however, indicated a clear arrest of the Δ21-mVDAC1 in the open state (Figure 3A), sharing similar characteristics with the Δ26-mVDAC1 deletion construct described by Abu-Hamad *et al*. [4]. An involvement thus of the N-terminal fragment in the maintenance of the overall integrity of the incorporated channel is highly plausible, as also demonstrated by NMR data [46], and cannot be restricted to stabilizing effects on the open conformation only. We suggest that flickering reflects rapid transitions between different sub-states of the channel, as we have observed noise reduction in bacterial porins engineered with a shorter-loop (unpublished data). Consequently, the observed flickering/noise may reflect complexities not solely attributed to the mobility of the N-terminal fragment, but also to other structural components of mVDAC1.

Under our BLM conditions, reconstituted mVDAC1 exhibited a 3.94±0.04 nS conductance in the open state (S0), consistent with previous observations, whereas the average conductance of the closed state was 2.34±0.06 nS, differing significantly from previously published values (Table 1) [3], [27], [39], [40], [41], [47]. Instead the closed state of mVDAC1 shows a broad distribution between two already previously recognized but not defined major states (S1, S2) [48]. In addition we find that the S2 state is itself split into two further sub-states S2A and S2B in a 2.4∶1 ratio. S2B exhibits a 1.48±0.02 nS conductance almost half of that of the S1 state (Figure 2C). Defining these main states and their transitions is essential not only for deciphering the channel's functionality, but also in order to acquire a platform for the characterization of VDAC variants with potentially subtle gating phenotypes. The latter may become pivotal when considering the VDAC-dependent mitochondrial metabolite transport.

Recently, Cheneke, van den Berg and Movileanu [49] analyzed the gating transitions of the three major open states of the OpdK channel from *Pseudomonas aeruginosa* and reported that the conformational fluctuations of the low amplitude current transitions stemmed from the highly flexible loop 7 occluding the pore. In VDACs however, the situation is less straight forward. Firstly, no major loop appears as a prominent candidate for channel occlusion, and secondly, although the N-terminal α-helix appears position-wise to exert control on the flux of metabolites [50], the latest NMR studies [51] revealed distinct mobility only for strands β2-β7. Here, the N-terminal helix appeared relatively rigid, raising doubts as to its involvement in controlling gating. Interestingly, and following our observations, the observed low amplitude current transitions of OpdK [49] mimic the mVDAC1 S1↔S2 transitions.

To further investigate the potential role of the N-terminal segment in voltage gating, we engineered and characterized double-cysteine mVDAC1 variants. Our cross-linked mVDAC1-A14C-S193C channel exhibited a clear preference for the open state (average dwell time 30.61±6.39 min ≡ 1836.60±372.91 s), attaining hence the S2 state with a strongly reduced probability (Figures 5B, 5C and S3). Having achieved the closed state, this variant failed to re-open. The oxidized and subsequently reconstituted mVDAC1-V3C-K119C variant, moreover, exhibited asymmetric gating characteristics (Figure 6), conducting in the open state at both negative and positive potentials, and closing only upon application of the reverse potential, depending on its orientation in the lipid bilayer (Figures 7A, 7B). Surprisingly, transitions between open and closed states were mediated through a series of conducting conformers, enabling final relaxation to an S2 conductance state. Open-to-closed state transitions occurred more slowly than the wild type, extending the average dwell time of the cross-linked channel in the S0 state to 78.58±3.99 s (Figures 7C, S3). A complete switch to native-like channel behavior was achieved following reduction of the cysteines of both variants by DTT (Figures 5A, 7A).

The conductance behavior of the cross-linked mVDAC1 variants shows that the choice of residues for covalent linking is crucial for 1) observing unique gating phenotypes and 2) achieving different immobilization degrees of the N-terminal α-helix. This is clearly reflected in the intensity of the gating phenotype of the mVDAC1-A14C-S193C variant. Affixing residue 14 on the barrel wall immobilizes the N-terminal fragment more effectively than affixing residue 3, almost locking the channel in its open conformation. Yet, the remaining flexibility of the mVDAC1-A14C-S193C channel, still allowed for an unfavourable and permanent closure to the S2 state. The oxidized mVDAC1-V3C-K119C variant on the other hand, although having lost its symmetrical gating, exhibited reversible S0-to-S2 transitions. In consequence, these findings suggest that the full flexibility of the N-terminal fragment is required in achieving transitions to and/or from the S1 state, and that S0-to-S2 can happen independently of S0-to-S1 transitions, probably through meta-stable intermediates of an alternative route (Figure 6). Latest studies identified an intrinsic asymmetric effect on the closure of VDAC depending on the sign of applied voltage [45]. This finding points to the existence of alternate mechanisms for channel closure. Although further experimentation is required to decipher the role of these events, it seems that the S0-to-S1 and S0-to-S2 transitions may represent separate closing modes, as hypothesized by Hiller and Wagner, and that S1 is not just an intermediate before complete relaxation of the channel in the S2 state.

*What information can be extracted from the closure of the cross-linked channels?* With respect to the A14C-S193C-mVDAC1 channel, fixing residue 14 to the porin's wall allows for mobility of residues 1–13 and the hinge region (KGFGYG). The arrest of the open state in this variant for ∼30 min (Figure 5C) indicates that residue 14 and the stretches around it participate in native channel opening. Nevertheless, the hinge region remains free to undergo conformational changes, finally causing the channel's closure. In doing so, it probably exerts mechanical strain on the relatively immobile α-helical part. We hypothesize that closure of this cross-linked channel is mediated by mechanical strain effects, stemming from the movement of the hinge region, which can further induce either a) the structural collapse of the barrel wall b) the irreversible unfolding of the N-terminal helix or c) a combination of the two. All scenarios could result in permanent channel closure as observed for the oxidized mutant.

**Figure 8:**
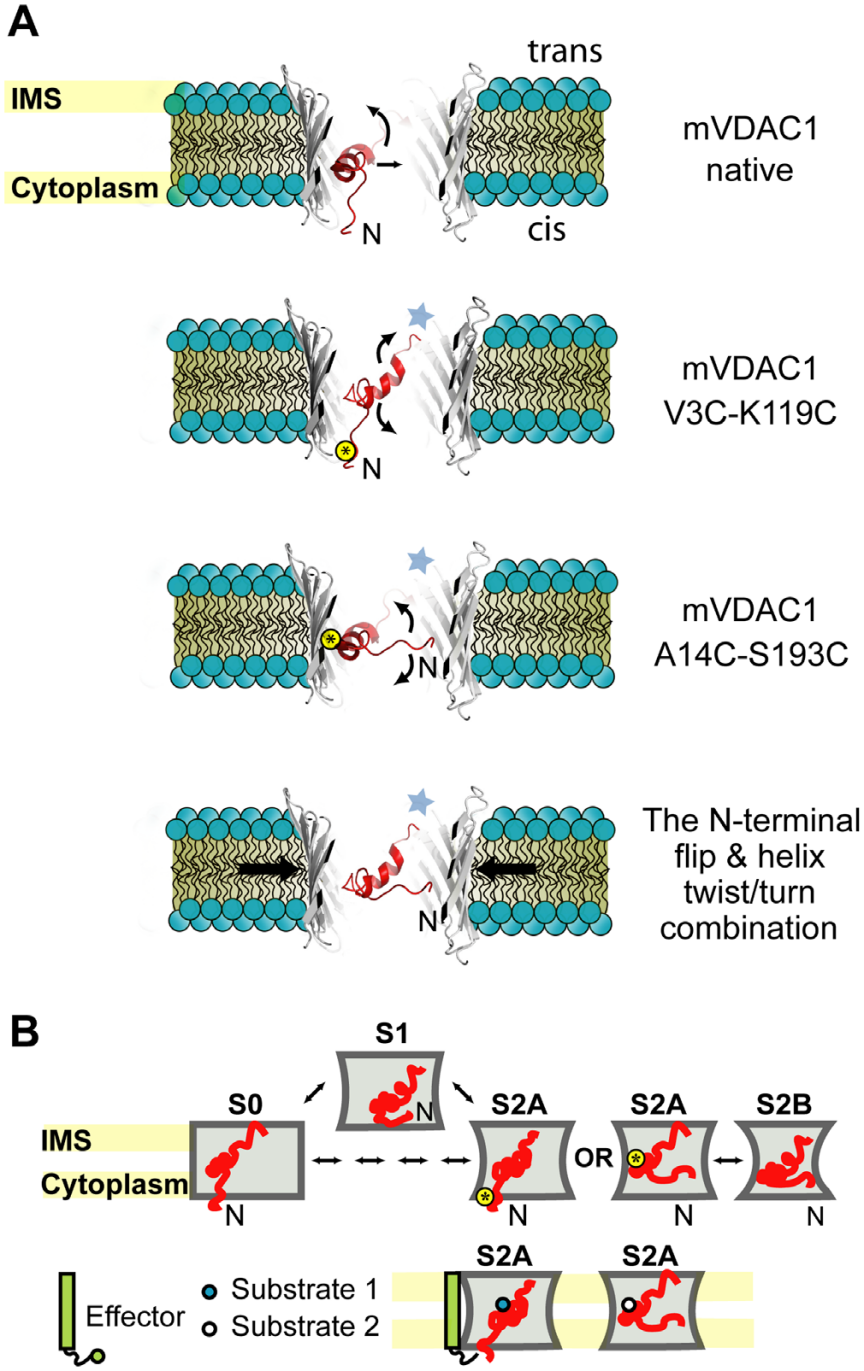
Native channel voltage gating involves the concurrent movement of the very N-terminus into the pore and induction of channel narrowing by partial wall collapse. A) Cross section of a 3D-model of mVDAC1, based on the 3EMN structure [3], parallel to the membrane plane. Cross-linking at the base and midpoint of the pore is indicated (yellow circled asterisk). Possible dynamic movements of the α-helix and the 1–4 residue N-terminal part are described for the two cross-linked mutants relative to the native channel. In the native channel, the N-terminal fragment is capable of both a flip and a twist/turn movement. The concurrence of these movements together with a constriction of diameter of the pore, close the channel. Cross-linking at the bottom (V3C-K119C) or the middle (A14C-S193C) of the pore, prevent the flip and twist/turn motions of the N-terminal fragment respectively. Thin arrows indicate possible directions of movement. Thick arrows indicate potential tightening of the pore diameter. The asterisk denotes movement contributions from the hinge region. Note that with the proposed orientation both N- and C- termini are cytoplasmic (*cis*) in accordance with McDonald *et al*. [56]. IMS; intra-membrane space (*trans*). B) N-terminal fragment movements in the context of the observed closed state conformations. In the open S0 state, the helix is located at the mid-point of the pore with the far N-terminus facing the cytoplasm (*cis*). Channel closure (S1 state) is initiated upon movement of the far N-terminus into the pore and is completed by the constricting movements of the pore’s wall. Oxidized mutants (yellow circled asterisk) become immobilized in an S2A state, exhibiting restricted N-terminal fragment flexibility and impede barrel wall movement. In the S2B state movements of both the barrel wall and the N-terminus complete channel closure. Effectors could control S1-to-S2 transitions enabling or prohibiting interactions with substrates thus regulating apoptotic responses. Footnote: While this manuscript was under review, Zachariae *et al*. [57] reported that the closure of voltage-dependent anion channels is largely regulated by the mobility of the β-barrel scaffold. These authors note however, that the mobility/flexibility of the N-terminal domain, albeit lower than that of the β-barrel, is also essential in shaping the channel’s gating characteristics. They also suggest that gating does not require the exit of the N-terminus from the inside of the channel, and show that minor structural reorganizations of the N-terminal α-helix, whilst still inside the pore, can have noticeable effects on the channel’s conducting capacity. Their observations are also in agreement with our proposed model of VDAC voltage-induced gating, where the conformational changes of the N-terminal fragment, in conjunction with dynamic movements of the barrel walls define transitions between specific conducting states. In essence, both works demonstrate the importance of the N-terminal fragment in a) the preservation of the overall integrity of the channel and b) the regulation of transitions/entry into states which, in combination with the dynamic movements of the barrel, eventually define the channel’s gating behavior.

In the V3C-K119C variant, where cross-linking at residue 3 allows for more N-terminal flexibility (extending from residue 4 to the hinge region), voltage gating lacks the symmetrical features of the native channel’s activity. This means that this cross-linked variant, although capable of conducting in the open state at both positive and negative potentials, closes only upon application of one. This indicates that only one direction of the applied electric field destabilizes the tethered N-terminal helix. Apparently, immobilization and/or reduced flexibility of the N-terminal segment make it less prone to unfolding by an applied electric field. Furthermore, the behavior of this channel upon potential reversal suggests that the residue 4–26 stretch still undergoes structural reorganizations allowing for slower S0-to-S2 transitions (Figures 6, 7C and S3). Interestingly, these structural changes can be reversed with the next reversal of applied potential. Thus, in contrast to the irreversible structural changes observed in the oxidized A14C-S193C variant, partial immobilization of the far N-terminal part allows for a voltage-driven reversible reorganization/refolding of the α-helix. Current studies [52] suggest that VDACs assume their open state upon N-terminal fragment detachment from the pore wall and subsequent translocation to the cytoplasmic side (“paddle” movement). The behavior of our cross-linked variants however indicated that, although a structural reorganization of the 1–14 residue segment is required for voltage gating, the so-called “paddle” movement is not required. Consequently, we propose that closure is mediated by the concurrence of 1) a flipping motion of the far N-terminal part into the middle of the channel and 2) a local unfolding/refolding event of the α-helix (Figure 8).

Recently, Teijido *et al*. [11] reported that the L10C-C127A-A170C-C232A-mVDAC1 mutant displayed native-like channel activity under oxidative conditions, and concluded that voltage gating did not involve and/or require rearrangements of the covalently linked N-terminal fragment. Contrary to that, both our mVDAC1 double cysteine variants showed under oxidative conditions distinct gating profiles, avoiding the S1 and relaxing only at the S2 state. The reasons for the observed deviant behavior of the L10C-A170C-mVDAC1 variant can be manifold. First, *in situ* oxidation as performed in [11] failed in our hands under the chosen BLM conditions and therefore required the usage of *in vitro* oxidized and purified double-cysteine mVDAC1 variants. Accordingly, proper disulfide bond formation in the used samples was verified by ESI-MS. Secondly, we only characterized mVDAC1 variants without a charged N-terminal affinity tag, thus avoiding possible gating interference. Finally, we restricted the number of single-channel incorporations to a minimum (≤5). Last but not least, and as previously indicated, the choice of disulfide-mediated fixing points is crucial for gating behavior and formation of arrested channels. For example, failure to completely immobilize the N-terminal helix results in native-channel behavior, whereas forcing the outward placement of the N-terminal region by generating cross-linked mVDAC1 homodimers via their N-termini [52] arrests gating like a deletion of the N-terminal helix itself.

As previously shown, channel closure provides a regulatory mechanism for control of substrate and/or ion efflux. In the 3EMN crystal structure, salt bridges engaging residues K12-D16-K20 are evident. Recent studies have identified K12 and K20 to be important selectivity determinants. Törnroth-Horsefield and Neutze [53], proposed that even a modest rotation of the N-terminal helix around the hinge region has the potential of both occluding the channel and altering its substrate-specificity. Thus it is tempting to hypothesize that a movement and a structural reorganization of the N-terminal segment (Figure 6A) is not only responsible for channel closure but also defines the channel’s selectivity through the exposure of charged residues. The permeability of mVDAC1 for succinate, citrate or inorganic phosphate ions was shown to be ∼10-fold lower than for chloride ions [2], [54]. Choudhary *et al*. also reported that the channel selectivity is determined by the orientation of the channel in the bilayer and that lower conductance states are anion selective [10]. Establishing thus the affinities of the low conducting states for specific anions becomes an essential step in understanding the regulation of mitochondrial biochemistry.

So far we have demonstrated that the dynamic character of the open-to-closed transitions of VDAC1 clearly depends on the flexibility of the N-terminal α-helix, and we have considered the effects of the different extents of immobilization of the helix on possible structural changes associated with those transitional events. With this study we prove the importance of the N-terminal fragment’s flexibility in controlling gating, and corroborate earlier hypotheses by Hiller and Wagner [9].

In summary, conformational restriction of the N-terminal α-helix dynamics strongly affects the gating characteristics of mVDAC1. Cross-linked mVDAC1 variants exhibit distinct open-to-closed state transitions, with omission of the S1 state (Figures 5B, 7B and S3). Different biological responses can be elicited through the regulation of these transitions triggered by the many effectors which interact with mVDAC1 and its N-terminus (Figure 8B), offering thus a control point in mitochondrial apoptosis. Interaction between mVDAC1 and the anti-apoptotic protein Bcl-x_L_ was recently shown to require the N-terminal mVDAC1 helix and resulted in a ∼2.5-fold reduction of the channel’s conductance [55]. Such a reduction would be consistent with occupancy of the S2 state in our model (Figure 8B). We are currently further investigating whether a local folding/unfolding of the α-helix controls the exposure of charged residues, contributing thus to the pore’s ion selectivity. In conclusion, although the N-terminal segment is not the sole determinant of the channel’s activity and selectivity [10], [51], it certainly plays an essential part, supported undoubtedly further by the structural plasticity of the barrel wall.

## Supporting Information

Figure S1 **Confirmation of disulphide formation in the A14C-S193C-mVDAC1 variant by mass spectrometry.** MALDI-MS tryptic peptide mass fingerprint of A14C-S193C-mVDAC1. The peaks of the obtained chromatograms reveal the ionization profile of two cross linked fragments for the 993.0–995.4 and 1489–1493 mass range (m/z). Detected masses for the +3 and +2 ionization states are labelled (red and green) and are compared to the expected masses in the table below. Calculations were performed using the Peptide Mass Calculator v3.2 (Jef Rozenski, 1999).
(TIF)

Figure S2 **Oxidation of reduced A14C-S193C-mVDAC1 over time.** A) Non reducing SDS-PAGE profile of DTT (10 mM) treated A14C-S193C-mVDAC1. Following the removal of the reducing agent, protein aliquots (2 µg) were taken over a period of 8 days, mixed with SDS-PAGE sample buffer without β-mercaptoethanol and heated at 95°C (5 min) prior to 12% SDS-PAGE electrophoresis. Spontaneous oxidation of cysteines was visible (increased electrophoretic mobility-Ox.) after the first day. Red.; reduced. B) Percentage decrease of the fully-reduced A14C-S193C-mVDAC1 population with time. Percentages were determined from the densitometric analysis of the SDS-PAGE in (A). The half life of the reduced species was ∼22 hours.
(TIF)

Figure S3 **Average dwell times of native and engineered mVDAC1 variants.** Native (*n* = 310) and cysteinless (*n* = 37) channels exhibited similar dwell times (*P* = 0.98) before making a transition to any state, whereas the dwell times of the oxidised cysteine-engineered variants (A14C-S193C; *n* = 14, V3C-K119C-initial potential; *n* = 8) were significantly extended with respect to the native protein (*P*<10^−4^ and *P*≤10^−4^ respectively). Note that after potential reversal the capacity of the oxidized-V3C-K119C channel (*n* = 24) to alternate between individual states mimics that of the native channel (*P* = 0.68).
(TIF)

Table S1 **Analysis of amplitude histograms fitted by Gaussian functions.**
(DOC)
