# Supplementary figures and images for: Flexibility of the N-Terminal mVDAC1 Segment Controls the Channel’s Gating Behavior
Source: PLoS One. 2012 Oct 23;7(10):e47938. doi: 10.1371/journal.pone.0047938 (PMC3479125; doi:10.1371/journal.pone.0047938)

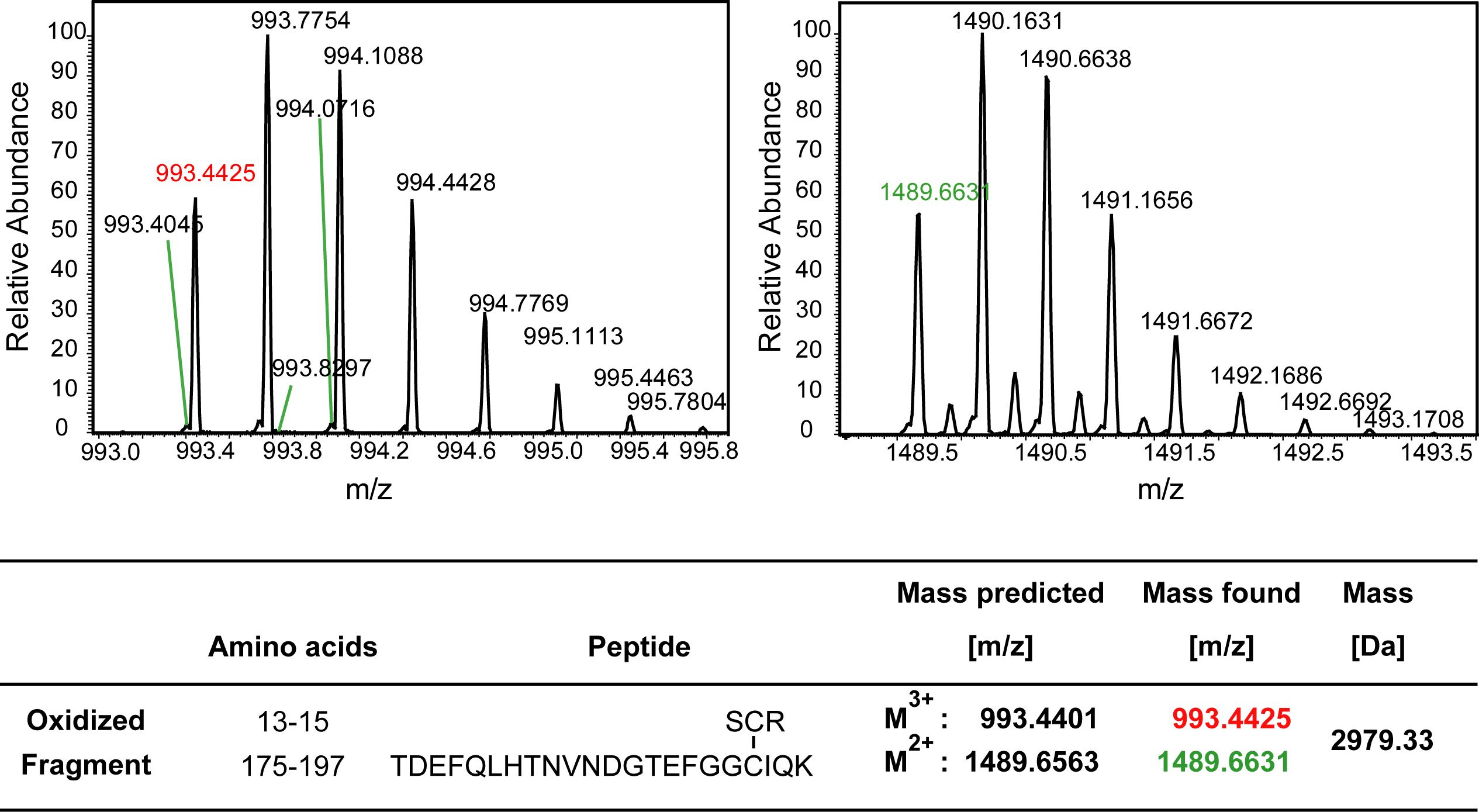

Supplement: Figure S1 — Confirmation of disulphide formation in the A14C-S193C-mVDAC1 variant by mass spectrometry. MALDI-MS tryptic peptide mass fingerprint of A14C-S193C-mVDAC1. The peaks of the obtained chromatograms reveal the ionization profile of two cross linked fragments for the 993.0–995.4 and 1489–1493 mass range (m/z). Detected masses for the +3 and +2 ionization states are labelled (red and green) and are compared to the expected masses in the table below. Calculations were performed using the Peptide Mass Calculator v3.2 (Jef Rozenski, 1999). (TIF) [file pone.0047938.s001.tif]

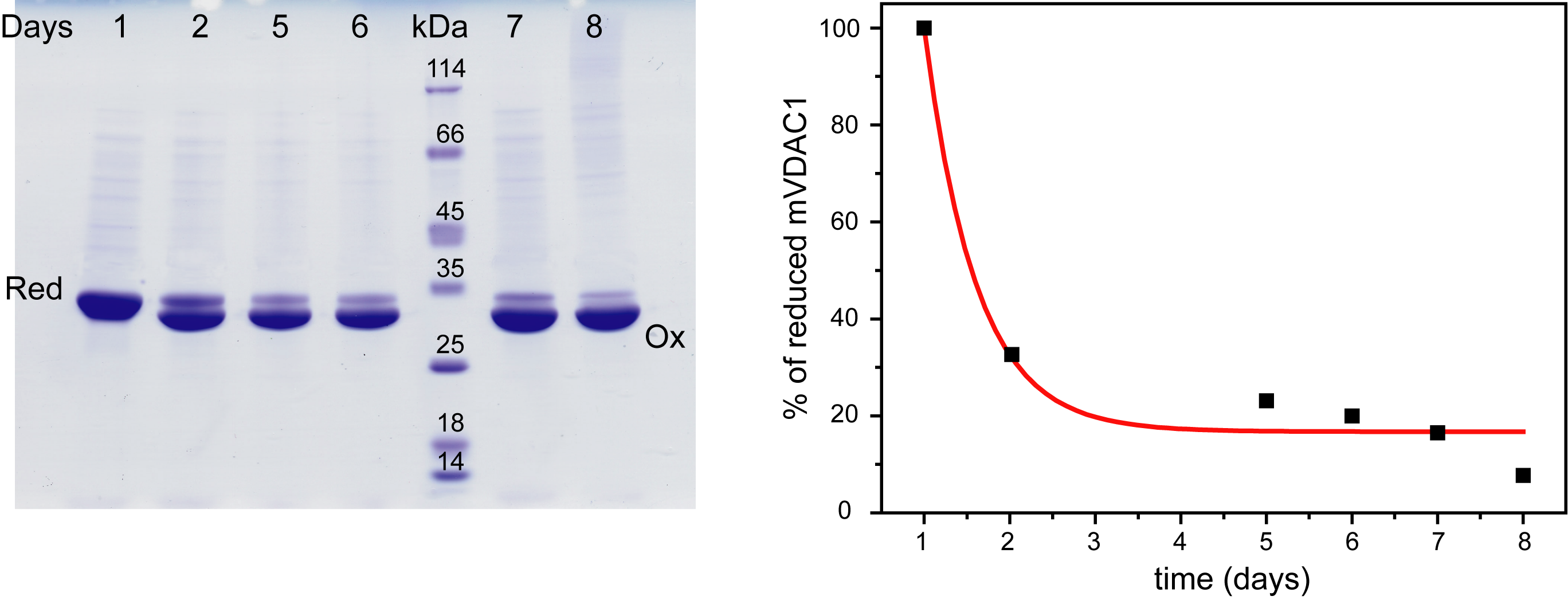

Supplement: Figure S2 — Oxidation of reduced A14C-S193C-mVDAC1 over time. A) Non reducing SDS-PAGE profile of DTT (10 mM) treated A14C-S193C-mVDAC1. Following the removal of the reducing agent, protein aliquots (2 µg) were taken over a period of 8 days, mixed with SDS-PAGE sample buffer without β-mercaptoethanol and heated at 95°C (5 min) prior to 12% SDS-PAGE electrophoresis. Spontaneous oxidation of cysteines was visible (increased electrophoretic mobility-Ox.) after the first day. Red.; reduced. B) Percentage decrease of the fully-reduced A14C-S193C-mVDAC1 population with time. Percentages were determined from the densitometric analysis of the SDS-PAGE in (A). The half life of the reduced species was ∼22 hours. (TIF) [file pone.0047938.s002.tif]

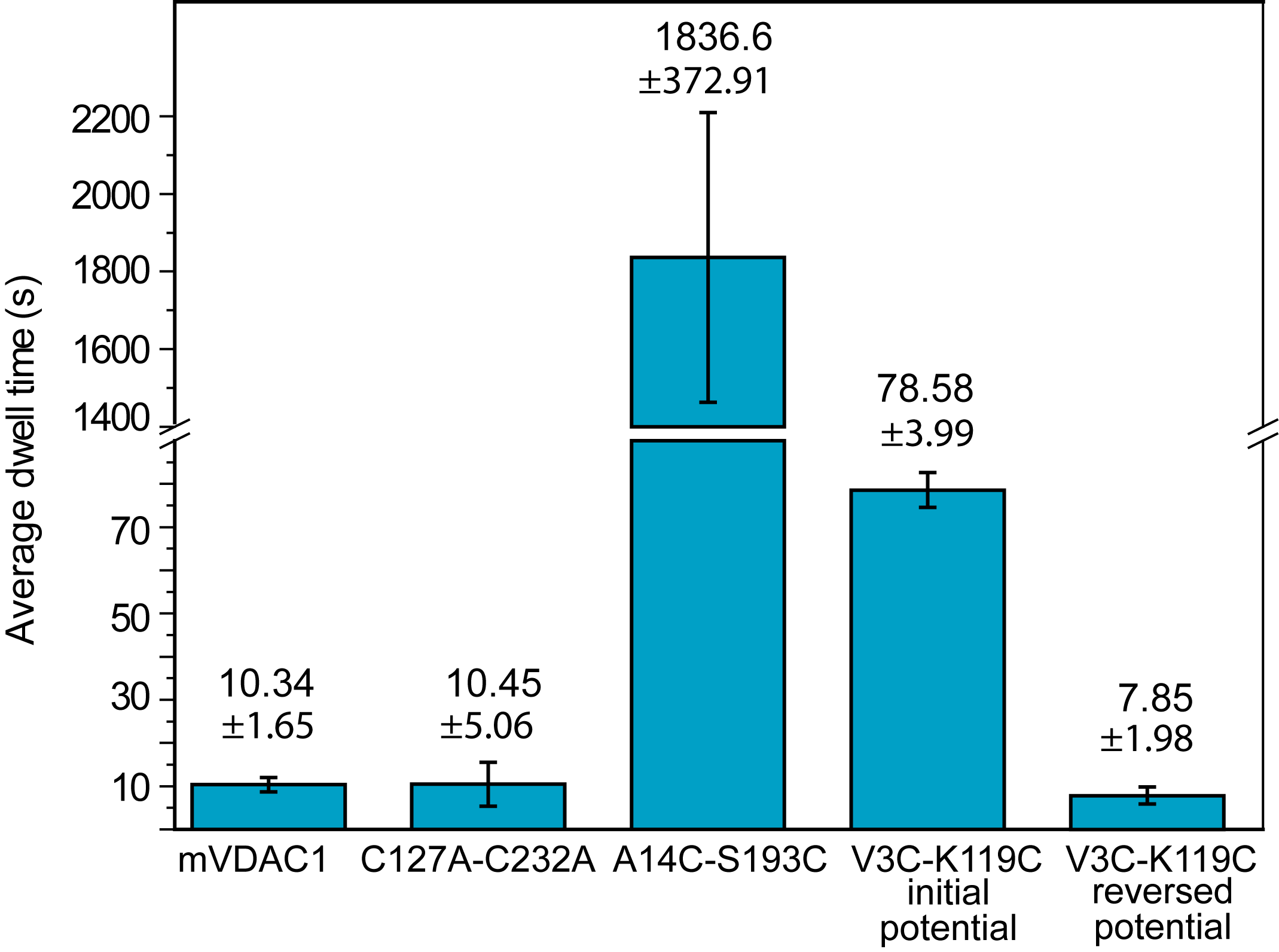

Supplement: Figure S3 — Average dwell times of native and engineered mVDAC1 variants. Native (n = 310) and cysteinless (n = 37) channels exhibited similar dwell times (P = 0.98) before making a transition to any state, whereas the dwell times of the oxidised cysteine-engineered variants (A14C-S193C; n = 14, V3C-K119C-initial potential; n = 8) were significantly extended with respect to the native protein (P<10−4 and P≤10−4 respectively). Note that after potential reversal the capacity of the oxidized-V3C-K119C channel (n = 24) to alternate between individual states mimics that of the native channel (P = 0.68). (TIF) [file pone.0047938.s003.tif]
