# Supplementary material for: Flexibility of the N-Terminal mVDAC1 Segment Controls the Channel’s Gating Behavior
Source: PLoS One. 2012 Oct 23;7(10):e47938. doi: 10.1371/journal.pone.0047938 (PMC3479125; doi:10.1371/journal.pone.0047938)
Supplement: Table S1 — Analysis of amplitude histograms fitted by Gaussian functions. (DOC) [file pone.0047938.s004.doc]

| **Statistical**  **Model** | **Amplitude** | **Mean**  **(nS)** | **STDV**  **(nS)** | ***R^2^*** |
| --- | --- | --- | --- | --- |
| 1. Single Gaussian  complete range | 73.70 ± 8.20 | 2.34 ± 0.06 (S1) | 0.57 ± 0.1 | 0.76 |
| 2. Single Gaussian  on S1 population | 79.69 ± 3.21 | 2.53 ± 0.02 (S1) | 0.32 ± 0.06 | 0.97 |
| 3. Single Gaussian  on S2 population | 79.11 ± 9.23 | 2.03 ± 0.03 (S2) | 0.25 ± 0.05 | 0.88 |
| 4. Sum of two Gaussians  complete range | 70.42 ± 9.35 (S1)  63.98 ± 10.77 (S2) | **2.61 ± 0.04(S1)**  **1.90 ± 0.06 (S2)** | 0.27 ± 0.08 (S1)  0.26 ± 0.08 (S2) | 0.91 |
| 5. Sum of two Gaussians  on S2 population | 80.49 ± 5.00 (S2A)  39.06 ± 7.94 (S2B) | 2.03 ± 0.01 (S2A)  1.48 ± 0.02 (S2B) | 0.19 ± 0.02 (S2A)  0.08 ± 0.02 (S2B) | 0.98 |

T-test between S1 means from 1 (*n* = 680) & 2 (*n* = 386): Different (*P* = 0.0193)

T-test between S1 means from 1 (*n* = 680) & 4 (*n* =680): Different (*P* = 0.0002)

T-test between S1 means from 2 (*n* = 386) & 4 (*n* =680): Similar (*P* = 0.1475)

T-test between S2 means from 3 (*n* = 294) & 4 (*n* =680): Similar (*P* = 0.1642)

T-test between S2 means from 3 (*n* = 294) & 5 (*n* =294): Same (*P* = 1.0000)

T-test between S2 means from 4 (*n* = 680) & 5 (*n* =294): Similar (*P* = 0.1558)

T-test between S1 mean from 4 (*n* = 386) & S2 from 4 (*n* = 294) : Different (P ≤10^-4^)

T-test between S2A mean from 5 (*n* = 224) & S2B from 5 (*n* = 70) : Different (*P* ≤10^-4^)
